# Three-Dimensional Microscopic Image Reconstruction Based on Structured Light Illumination

**DOI:** 10.3390/s21186097

**Published:** 2021-09-11

**Authors:** Taichu Shi, Yang Qi, Cheng Zhu, Ying Tang, Ben Wu

**Affiliations:** 1 Department of Electrical and Computer Engineering, Rowan University, 201 Mullica Hill Rd., Glassboro, NJ 08028, USA; shitai32@students.rowan.edu (T.S.); qiy1@students.rowan.edu (Y.Q.); tang@rowan.edu (Y.T.); 2 Department of Civil and Environmental Engineering, Rowan University, 201 Mullica Hill Rd., Glassboro, NJ 08028, USA; zhuc@rowan.edu

**Keywords:** 3D reconstruction, microscopy imaging, structured light imaging

## Abstract

In this paper, we propose and experimentally demonstrate a three-dimensional (3D) microscopic system that reconstructs a 3D image based on structured light illumination. The spatial pattern of the structured light changes according to the profile of the object, and by measuring the change, a 3D image of the object is reconstructed. The structured light is generated with a digital micro-mirror device (DMD), which controls the structured light pattern to change in a kHz rate and enables the system to record the 3D information in real time. The working distance of the imaging system is 9 cm at a resolution of 20 μm. The resolution, working distance, and real-time 3D imaging enable the system to be applied in bridge and road crack examinations, and structure fault detection of transportation infrastructures.

## 1. Introduction

Three-dimensional (3D) microscopic imaging is a key tool for structure fault detection and analysis of the material degradation in transportation infrastructures [1,2]. The 3D test results provide valuable information of transportation infrastructure, such as the depth and width of a crack, and the volume of an abnormal protrusion [3,4]. Based on scanning methods, the 3D microscopic systems have been widely studied and achieve a high lateral resolution (hereinafter referred to as resolution). By controlling and scanning the focal point in the x, y and z directions [3,4], the scanning microscopes capture the object information at each scanned point. The scanning methods remove the interference signals from adjacent points to achieve a resolution higher than the diffraction limit. The high resolution is ideal for a lab test, whereas for a field test in the transformation system, the test speed becomes the major factor to be considered. The scanning methods require seconds or minutes to finish a scan and obtain a 3D image. The long imaging time requires the sample to be held stable during the imaging process, which is not ideal for field tests.

Three-dimensional imaging based on structured light illumination has been used to reconstruct 3D images in relatively large scales, such as face recognition. A widely used structured light pattern is black–white ray stripes and is generated by the averaging four-frame shifting method [5]. After the structured light is projected onto the surface of the object to be measured, a camera system captures the object image from a different angle of view. The height information and 3D morphology of the object surface create deformations of the structured light. To quantitatively measure the deformation, the system is calibrated with pre-known patterns, and the relative positions between the object, the projector and the camera are determined in the calibration process. By calculating the deformation of the stripes and the relative positions between the projector and camera, the 3D information of the object is measured [6,7,8]. A calibration algorithm has recently been proposed by G. Taubin et al. [6], which significantly reduces computation time and improves the resolution of the imaging system.

In this paper, we applied the structured light illumination to microscopy and developed an ultra-fast 3D microscopic imaging system. A digital micro-mirror device (DMD) was used to generate the structured light [9]. The structured light was then projected on to the object through a microscopic system. To the best our knowledge, the coupling of structured light to a microscopic system for 3D image reconstruction has not been widely used. Another microscopic system was used to image the object on a charge-coupled device (CCD). Both the projection system and the imaging system were designed and optimized for the field test of transportation infrastructure. The imaging and reconstruction times were less than one second, which meets the needs of an instant measurement for a field test. The physical imaging setup allowed the traditional computer vision 3D reconstruction algorithm to be applied in the microscopic image processing [10,11,12].

## 2. Methods

The principle of 3D imaging based on structured light illumination is to calculate the depth information by observing the deformation caused by the uneven surface of the object. The structured light is generated by a phase-shifting method [5,13,14]. With the relative positions between the camera and projector, the depth information of the 3D object is calculated by the phase-shift of the structured light [15,16,17,18]. Image reconstruction using a microscopic system is different from image reconstruction that uses a traditional large-scale camera system in two aspects, and a redesign of both the reconstruction algorithm and the hardware system is needed to achieve an ideal resolution.

First, the intensity fluctuations of the structured light cause an error of phase-shifting. The impact of such fluctuations is negligible in large-scale imaging, whereas in microscopic systems, a relatively small light–power fluctuation causes numerous errors that cannot be neglected. This is caused by the fact that the microscopic systems use a larger aperture compared with the apertures of most large-scale imaging systems. The large aperture leads to a large aberration, and the aberration causes reconstruction errors. If the projection system uses black and white stripes with the same width, the observed white stripes (bright area) are significantly larger than the black stripes (dark area). The boundary is also blurred from the impact of the aberration. The change in white stripes and the blurred boundary is based on the optical transfer functions of the microscopic projection system and the imaging system. We redesigned the patterns of the structured light and optimized the threshold function that identifies the boundary of black and white stripes based on the optical transfer functions.

Second, the field depth of the microscopic systems is much smaller than the field depth of the camera systems used for large-scale imaging. To achieve a high resolution and to enable adequate illuminance, the microscopic systems are designed with large apertures, and the field depths are inversely proportional to the radius of the aperture. For 2D imaging, the limited field depth blurs the image when the object does not overlap with the focus plane of the imaging system. For 3D imaging, the limited field depth affects both the imaging sub-system and the projection sub-system. The resolution of the 3D reconstruction system depends on the field depths of both sub-systems. We designed the system by optimizing the position of the object, the focus points of the two sub-systems and the angle between the two sub-systems to maximize the overall field depth and the resolution.

**Figure 1:**
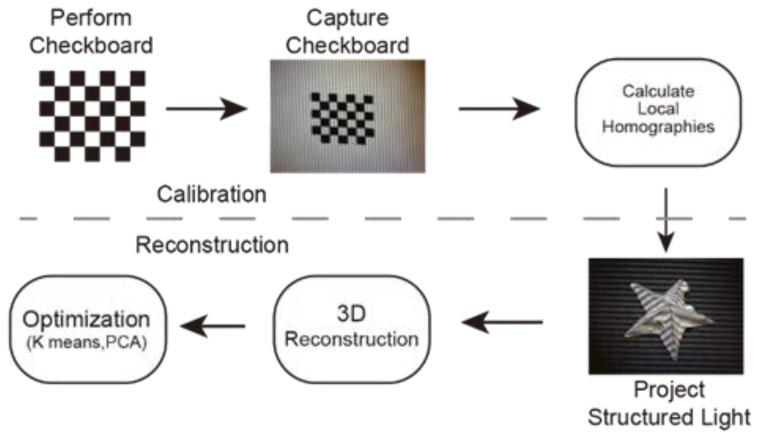
Reconstruction flow map.

Figure 1 shows the flow chart of the reconstruction system, which includes two major steps, calibration and reconstruction. A checkboard with a pre-known dimension is used to calibrate the system. The checkboard is a surface with black and white square lattices. The system is calibrated by changing the position and angle of the checkboard surface. At least three different positions are used to perform the calibration. The calibration process measures the relative positions between the camera and the projector, and once the calibration process is finished, the system can test different objects without the need for re-calibration. The second step is the reconstruction; here, the testing object is illuminated with structured light and the 3D profile of the object is reconstructed based on the deformation of the structured light and the relative positions between the projection system and the imaging system. After reconstruction, the reconstructed image is further processed to minimize measurement errors.

The calibration includes three sub-steps (Figure 1). The checkboard is placed in three different positions, all of which are within the depth of fields of both the imaging system and the projection system. The images of the checkboard are compared with the pre-stored information of the checkboard to obtain the transformation information [6]. The transformation information can be represented by the transfer matrix. The transfer matrix M is shown in the following equation and transfers the projector space to the camera space. A point v in the projector space can be represented by $v={\left[x,y,z\right]}^{T}$, and its corresponding coordinate in the camera space is $u={\left[\;x^{\prime },\;y^{\prime },\;z^{\prime },1\right]}^{T}$, and v, u and M satisfy the following equation:

(1) $$\left[\begin{array}{c} x\\ y\\ z \end{array}\right]=M\left[\begin{array}{c} \begin{array}{c} x^{\prime }\\ y^{\prime } \end{array}\\ \begin{array}{c} z^{\prime }\\ 1 \end{array} \end{array}\right],\;M=\left[\begin{array}{ccc} r_{11} & r_{12} & \begin{array}{cc} r_{13} & t_{x} \end{array}\\ r_{21} & r_{22} & \begin{array}{cc} r_{23} & t_{y} \end{array}\\ r_{31} & r_{32} & \begin{array}{cc} r_{33} & t_{z} \end{array} \end{array}\right]$$

M can be written in the form of M=K[R|T], where R shows the rotation of the coordinate and T shows the translation of the coordinate. Both are an extrinsic parameter matrix. We also need an intrinsic parameter matrix:

(2) $$K=\left[\begin{array}{ccc} f_{x} & s & x_{0}\\ 0 & f_{y} & y_{0}\\ 0 & 0 & 1 \end{array}\right]$$

In this matrix, $f_{x}$ and $f_{y}$ are the distances between the focal planes to the viewpoint. The central point of the projector is $\left(x_{0},y_{0}\right)$. The s is a twist parameter, which was 0 in our experiment. We calculated local homographs [11,18,19,20,21] in the experiment. Suppose a corner on the checkboard $q={\left[\mathrm{col},\mathrm{row},1\right]}^{T}$, which is captured by the camera, then the corresponding point on the captured image is $p={\left[x,y,1\right]}^{T}$, then we find the homography $\hat{H}$ that minimizes:

(3) $$\hat{H}=\underset{H}{\mathrm{argmin}}\underset{\forall p}{\sum }{\left\|q-\mathrm{Hp}\right\|}^{2}$$

The microscopic imaging system inverses the image, so the point pair [q(n),p(n)] becomes [q(n),p(N−n)]. Thus, for each point on the checkboard:

(4) $$q\left(n\right)=\hat{H}p\left(N-n\right)$$

The transformation matrix with inverted image can be represented by $M^{\prime }$:

(5) $$M^{\prime }=\;\left[\begin{array}{ccc} -r_{11} & -r_{13} & -\begin{array}{cc} r_{12} & t_{x} \end{array}\\ r_{21} & r_{23} & \begin{array}{cc} r_{22} & t_{y} \end{array}\\ r_{31} & r_{33} & \begin{array}{cc} r_{32} & t_{z} \end{array} \end{array}\right]$$

With the pre-known information about the checkboard, and the calibration, $M^{\prime }$ can be measured.

## 3. Experimental Setup

**Figure 2:**
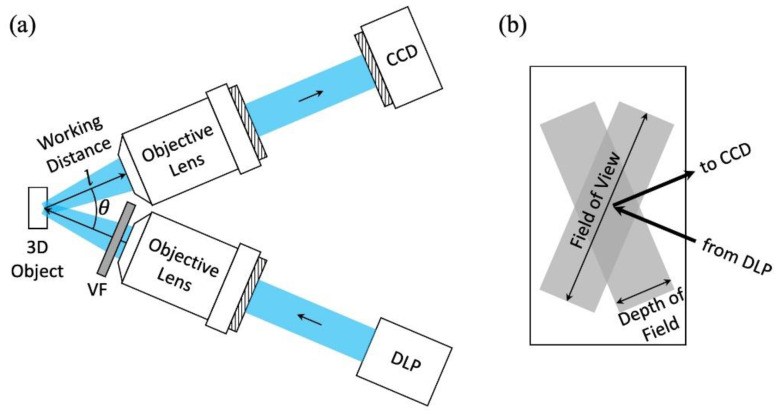
(**a**) Experimental setup. CCD, charge-coupled device camera; VF, variable neutral density filter; DLP, digital light processing projector. (**b**) Enlarged view of the rectangle region labeled with “3D object” in (**a**).

The experimental setup of the 3D microscopic image reconstruction system is shown in Figure 2a. The top is the imaging sub-system, and the bottom is the projection sub-system. A DLP generated the structured light, which was coupled to the object plane through a microscopic objective lens. The structured light included horizontal and vertical stripes, indicated by black and white patterns. With different densities and different widths of the stripes, both the overall profile and details of the 3D object were obtained. A 3D image was reconstructed with 42 patterns, with 21 horizontal-stripe patterns and 21 vertical-stripe patterns. The resolution of the system depended on the finest pattern, which included 480 vertical or horizontal stripes.

A variable neutral density filter was placed between the projector and the object. The filter controlled the intensity of the structured light, so the light intensity was neither too strong to saturate the CCD camera nor too weak to obtain a clear image. The checkboard for calibration included 6 × 7 square lattices and the side of each square was 0.7 mm. The angle between the object lens of the imaging system and the object lens of the projection system (θ in Figure 2a) was optimized based on three factors: (1) Field of view and depth of field. Figure 2b is the enlarged view of the focus area in Figure 2a. The two grey rectangles show the focus region of the projection system and the imaging system. The long sides of the grey rectangles show the field of views, and the short sides of the grey rectangles show the depth of the fields. To capture the image of the object in a single reconstruction, the object was within the field of views of both the projecting system and imaging system, and to obtain a clear image, the 3D object was within the depth of fields of both the projection system and the imaging system (in the overlap region of the two grey rectangles). A smaller θ corresponds to larger overlaps of both the field of views and the depth of fields of the two systems; (2) Resolution of the depth. The depth information of the 3D object was obtained based on the fact that the projection system and the imaging system focused on the object from different angles, where the difference of the angle is θ. The larger θ corresponds to a larger resolution of the depth that the system can achieve; (3) Shape of the objective lens. The selection of the θ was also limited by the radius of the objective lens (r) and the working distance (l) (Figure 2a), where θ/2 has to be larger than arctan (r/l) to avoid contact between the two objective lenses. Under all these considerations, the optimized θ in the system was selected to be 31 degrees.

The DLP used in the experiment was the DLP 3010 Light Control Evaluation Module from Texas Instruments. The resolution of the DLP was 608 ∗ 684 pixels. The CCD in the experiment was a Dino-Lite Edge 3.0 digital microscope. The resolution of the CCD was 2560 ∗ 1440 pixels. The objective lens of the DLP sub-system provided a total magnification of 20× with a numerical aperture of 0.25. The objective lens of the CCD sub-system provided a total magnification of 2× with a numerical aperture of 0.1.

## 4. Results and Analysis

### 4.1. System Demonstration

**Figure 3:**
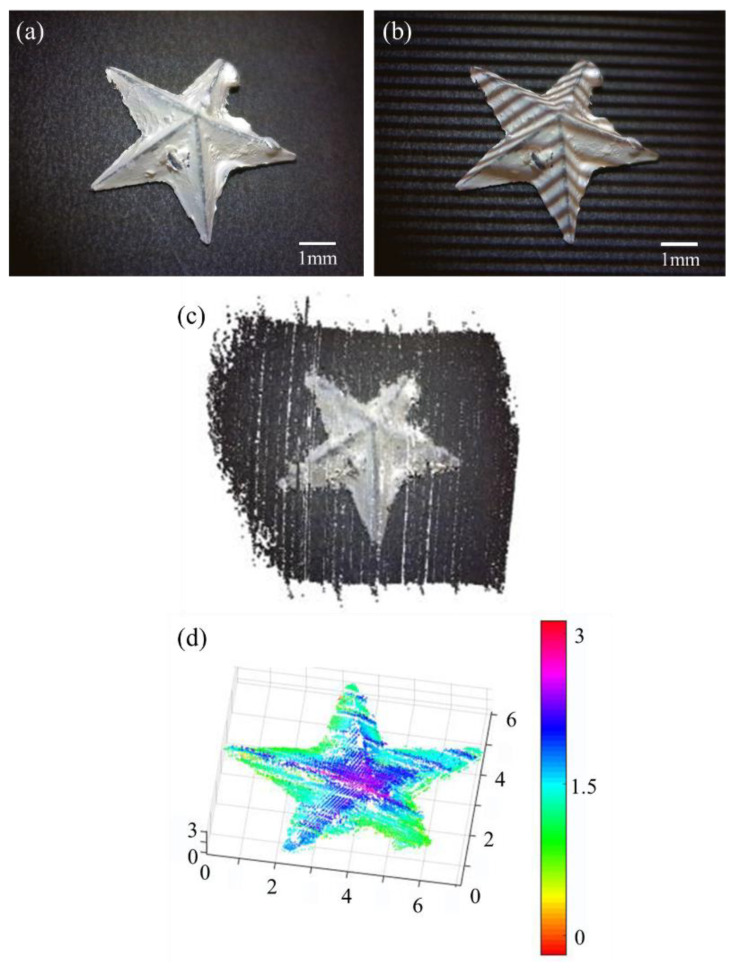
Experimental results of a 3D printed star. (**a**) Original 2D image of the star. (**b**) Image of the star illuminated with structured light. (**c**) Reconstruction point cloud of the star. (**d**) Heatmap that shows the depth and height information of the star. The x, y and z scales are in the unit of millimeters.

The reconstruction results are shown in Figure 3. The object under test was a 3D printed star with a diameter of 7 mm and height of 2.5 mm. Figure 3a is a 2D image of the star. Figure 3b shows the star illuminated with structured light. Figure 3c shows the reconstructed 3D point cloud. Figure 3d shows the results after optimization, and the heatmap shows the height information of the star. The optimization process used a K-means clustering algorithm to remove the background points and independent points.

The 3D profile of the object changes the patterns of the structured light. When structured light with a large density is used (for example, 480 stripes in Section 3), the stripes can be compressed by the 3D profile of the object. If the density of the compressed stripes reaches the resolution limit of the imaging system, the system cannot identify the stripes. Under such conditions, the algorithm is designed to reconstruct the image in the local region with a stripe density in the next level (480 stripes per picture to 240 stripes per picture). The reduced density corresponds to a reduced reconstructed resolution and can be improved by viewing the object from a different angle. Table 1 shows the vertical structured light patterns used in the experiment. The horizontal structured light patterns are the same. The two patterns in each row are complementary patterns. For example, pattern 5 has one white stripe and two black stripes, and pattern 6 has one black stripe and two white stripes. From pattern 11 to pattern 22, the stripe density in each group is twice the density of that in the previous group.

### 4.2. Resolution and Depth of Field

**Figure 4:**
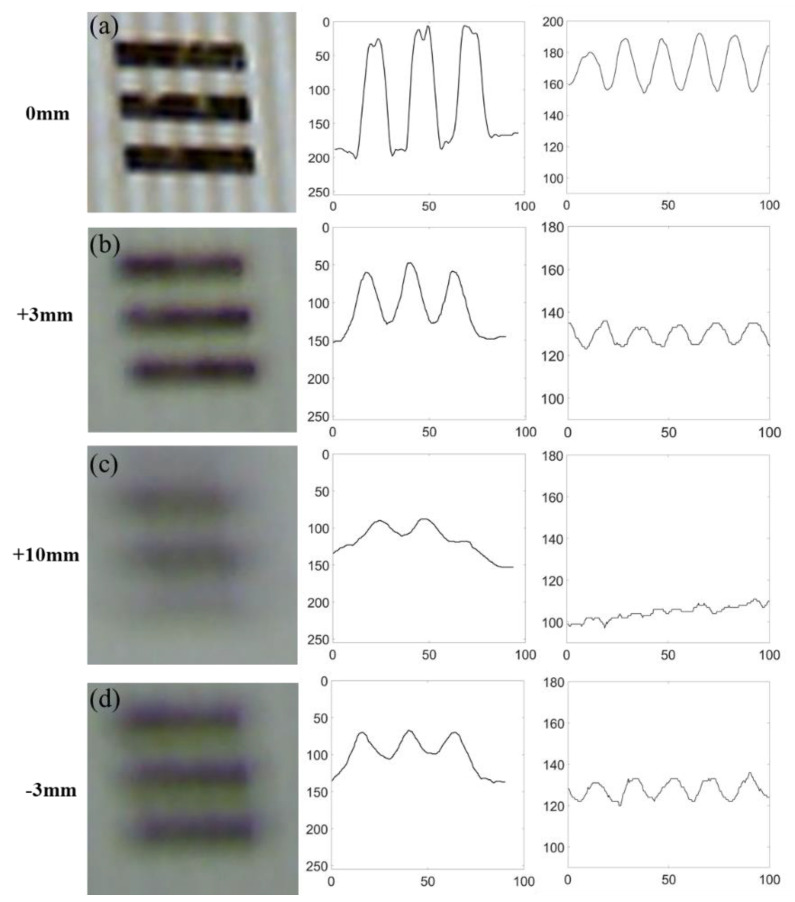
Resolution of the system at different positions. The four figures on the left show the image captured by the CCD camera. The four figures in the middle show the contrast of the horizonal resolution test chart. The four figures on the right show the contrast of the structured light with vertical stripes. For all four figures in the middle, and four figures on the right, the horizontal axes show the distance with units of micrometers, and the vertical axes show the 8-bit grey scale (0−255). Zero indicates that the test chart overlaps with the focus points of both the imaging system and the projection system (**a**). Positive sign indicates that the test chart moves away from the system ((**b**,**c**)) and negative sign means the test chart moves towards the system (**d**).

The resolution test chart with the 1951 USAF standard was used to test the lateral resolution and the depth of field of the reconstruction system [22]. The resolution here refers specifically to the lateral resolution. The depth of field of the system depends on both the projection sub-system and the imaging sub-system. When the test chart overlapped with both the focus points of the projection sub-system and the imaging sub-system, both the test charts (horizontal black bars in Figure 4a and the structured light (vertical strips in Figure 4a)) are clearly seen. The test chart in Figure 4 is group 5, element 2 in the 1951 USAF standard, with a line width of 15 μm.

Figure 4b–d show the resolution at different depths. The threshold contrast in the experiment was 0.02. The positive sign indicates the object moves towards the objective lens, and the negative sign indicates the object moves away from the objective lens. In Figure 4b,d, where the test chart is ±3 mm from the focus point, both the test chart and the structured light can be identified. The contrast of the structured light is enhanced with local integration (right four figures). In Figure 4c, where the test chart is 10 cm from the focus point, neither the test chart nor the structured light can be identified. The experimental results show that the system achieved a resolution of 20 μm with a depth of field that is ±3 mm.

## 5. Applications in Transportation Infrastructure Measurement

The 3D microscopic image reconstruction system fills a gap of the existing imaging methods for transportation infrastructures. In the construction of transportation infrastructure, measuring the change in soil water content in the natural environment is an important link to ensure the stability of infrastructure construction such as roads and bridges. Usually, imaging techniques are used to quantify soil samples for further research. Two-dimensional imaging methods have been widely studied in both the large-scale and microscopic scale and have been implemented in both lab tests and field tests [23,24,25]. Instant 3D imaging has been applied in large-scale image reconstruction, whereas instant 3D imaging in the microscopic scale has not yet been studied fully [26,27]. The 3D reconstruction system in this paper achieved an instant 3D image reconstruction at the microscopic scale and can be deployed in the following applications.

### 5.1. Volume Calcuation Based on a 3D Profile

**Figure 5:**
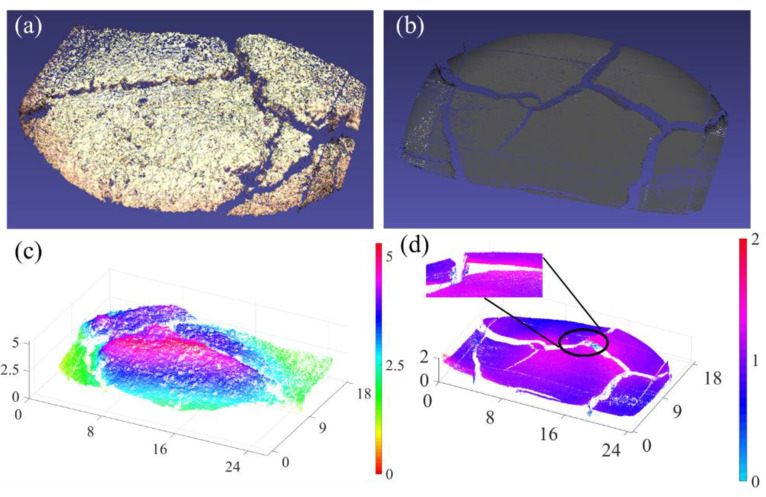
Reconstruction of soil samples. (**a**) Point cloud reconstruction of higher elevation samples. (**b**) Point cloud reconstruction of lower elevation samples. (**c**) Visualized data that show the depth and height information of the higher elevation samples. (**d**) Visualized data that show the depth and height information of the lower elevation samples. Black circle shows the effect of soil surface curling (Red points). The x, y and z scales are in the unit of millimeters.

Volume is an indispensable parameter to measure the abnormal structural variation in transportation infrastructures. The volumes of cracks or protrusions reflect the internal stress and the potential damage to the structure [28,29]. One of the influencing factors is a curling of the soil surface. In the process of soil turning from moist to dry, the soil will crack, and the surface will curl around the cracks. These soil surface curls will accelerate soil loss and increase the roughness of the soil surface. These curls will also block more wind and accelerate the destruction of the soil structure, which becomes a potential threat to sandstorms. These soil surface curls cannot be quantified with traditional 2D imaging techniques since depth information is not provided. Three-dimensional imaging technology, especially small-scale 3D reconstruction, can provide additional details about soil surface curling. Compared with 2D imaging technology, 3D imaging can provide more information about the volume changes during the soil drying process. Figure 5 shows the reconstructions of soil samples.

As shown in Figure 5d, the red part of the soil sample becomes higher. This is because as the soil dries, the edges of the cracks will curl upwards. By using only the 2D imaging method, the estimated volume of surface curling is not accurate. Such an effect is so small (within 1 mm) that it is difficult to quantify by a 2D top view or cross-sectional view. By using the 3D microscopic method, the volume of the cracks or protrusions can be accurately calculated based on the reconstructed 3D profile with a resolution of 20 μm. The 3D microscopic method can present an accurate reconstruction regardless of the elevation. The field of view of the microscopic system is 10 mm × 10 mm. If the object to be observed is larger than this area, the system can be mounted in a mechanical scanning stage to achieve a large area scan without a loss of resolution.

### 5.2. Time-Dependent Measurement

The time-dependent measurement indicates the changing trends of the structure. The system can measure time-dependent changes in both short time-frames and long time frames. For the short time-frame measurement, the reconstruction method can capture the instant changes in the time frame of sub-seconds, which is essential to observe the interaction between the microorganism and manmade materials, such as asphalt and concrete. By taking a 3D image at every second, a video can be created, and the 3D profile of both the microorganism and manmade materials are recorded in real time. For a long time-frame measurement, the system can perform a measurement in every few hours for the same target and record the changes of the target over the time-frame of months or years. Within such durations, the measured results can be used to analyze the performance of the transportation infrastructure under different weather conditions [30,31], the change in the external profile induced by internal stress [32,33], and the anti-fatigue performance [34,35].

### 5.3. Field Test with Long Working Distances

The long working distance enabled the system to perform a field test. The working distances of both the imaging objective lens and the projection objective lens (l in Figure 2a) were 10cm. Based on the analysis in Section 3, the θ was chosen to be 31 degrees. The working distance in the direction that is perpendicular to the object surface was lcos(θ/2), and considering the radius of the objective lens and the thickness of the filter, the effective working distance was 9 cm. Such a working distance enabled non-contact measurements. For a large area scan (Section 5.1), the long working distance also avoided the collision between the scanning microscope and the sample could be measured. Both the long working distance and instant image reconstruction properties enabled the system to perform a field test. The measurement can be performed directly on the surface of targets such as roads and bridges, without the need for sample preparation.

## 6. Conclusions

We proposed and experimentally demonstrated a 3D microscopic image reconstruction method based on structured light illumination. The system reached a resolution of 20 μm, with an imaging time of less than one second. The depth of field of the system was 6mm, and the working distance was 9 cm. The resolution, depth of field and working distance met the requirements of a field test for road crack examinations and structure fault detections. The system measured the 3D profile with a resolution of 20 μm, which can be used to accurately calculate the volume of a crack or an abnormal protrusion. The large working distance enabled a non-contact measurement, which is ideal for field tests.

## Figures and Tables

**Table 1:** Structured light pattern.

Pattern Number	Number of Stripes
1–2	All white or all black
3–4	2
5–6	3
7–8	5
9–10	9
11–12	15
13–14	30
15–16	60
17–18	120
19–20	240
21–22	480

## Data Availability

Not applicable.
